# Metachronous extraskeletal (soft tissue) epithelioid osteogenic sarcoma: a case report

**DOI:** 10.1186/s13256-019-2070-3

**Published:** 2019-05-09

**Authors:** Gireesha Rawal, Charanjeet Ahluwalia, Amit Kumar Yadav, Rashmi Arora

**Affiliations:** Department of Pathology, Vardhman Mahavir Medical College & Safdarjung Hospital, New Delhi, 110029 India

**Keywords:** Metachronous, Epithelioid, Osteosarcoma

## Abstract

**Background:**

Metachronous osteosarcoma is a rare form of osteosarcoma. The occurrence of metachronous tumor in soft tissue is exceedingly rare. The pathogenesis of metachronous osteosarcoma, as to whether it represents multiple true primaries or metastatic disease, is still obscure.

**Case presentation:**

A 49-year-old Indian man presented with progressively increasing swelling in his left hand of 2 months’ duration. An X-ray showed a soft tissue lesion. Contrast-enhanced computed tomography showed a soft tissue mass lesion, with peripheral enhancement and central necrotic areas in radial palmar soft tissue overlying second metacarpophalangeal region with no obvious bony osteolysis. Possibilities of acute abscess, resolving hematoma, or aggressive soft tissue mass lesion were suggested. An incision biopsy showed morphological features of epithelioid osteosarcoma, which was confirmed on immunohistochemistry. A detailed history revealed that our patient was diagnosed as having osteosarcoma of his right leg 3 years previously. Based on history, radiology, morphology, and immunohistochemistry, a final diagnosis of extraskeletal (soft tissue) epithelioid osteogenic sarcoma of the left hand occurring as a metachronous tumor 3 years after diagnosis of primary osteosarcoma in the right leg was given.

**Conclusion:**

This is probably the first reported case of extraskeletal (soft tissue) epithelioid osteosarcoma occurring as a metachronous tumor 3 years after diagnosis of primary osteosarcoma in the right leg. The prognosis of metachronous skeletal osteosarcoma is poor as compared to that of relapse limited to lungs. In late metachronous osteosarcoma, combined-modality therapy comprising surgery and aggressive chemotherapy may affect long-term survival. Lifelong follow-up of surviving patients with osteosarcoma is necessary and if metachronous osteosarcoma is discovered, it should be treated with curative intent.

**Electronic supplementary material:**

The online version of this article (https://doi.org/10.1186/s13256-019-2070-3) contains supplementary material, which is available to authorized users.

## Background

Excluding hematopoietic malignancies, osteosarcoma is the most common primary malignant tumor of bone [1], usually affecting metaphysis of long bones during maximum bone growth period, that is, between 10 and 20 years of age [2].

Metachronous osteosarcoma (presence of more than one primary osteosarcoma detected consecutively in a single person after a set time interval of > 6 months) is an uncommon form of osteosarcoma; its occurrence in soft tissue is rare, with almost all reported cases being at bony sites [3]. Its incidence is reported as 1–10% of all cases of osteosarcoma [4]. Extraskeletal osteosarcoma accounts for approximately 1% of soft tissue sarcomas and 4% of osteogenic sarcomas [5]. Contrary to conventional osteosarcoma, its extraskeletal counterpart more commonly affects adults (> 40 years) [6], with the lower extremity being the most common location [6]. It usually demonstrates a central pattern of ossification which shows contrast enhancement on magnetic resonance imaging (MRI) and positron emission tomography (PET) avidity. On histology, this variant mimics its conventional subtype [5]. Treatment modalities include primary surgery, multi-agent chemotherapy (both neoadjuvant as well as adjuvant), and radiotherapy (palliative as well as adjuvant). Chemotherapy protocols include high-dose methotrexate (HD-MTX), doxorubicin, cisplatin, and ifosfamide [7].

The pathogenesis of metachronous osteosarcoma, as to whether it represents multiple true primaries or metastatic disease, is still obscure. However, metachronous osteosarcoma has a clinical significance in that it is a potentially curable disease [8]. Karyotyping to reveal a possible clonal relationship between these tumors may help in estimating prognosis and guiding therapy intensiveness [9]. We present here a case of metachronous osteosarcoma because of its unusual presentation.

## Case presentation

**Fig. 1:**
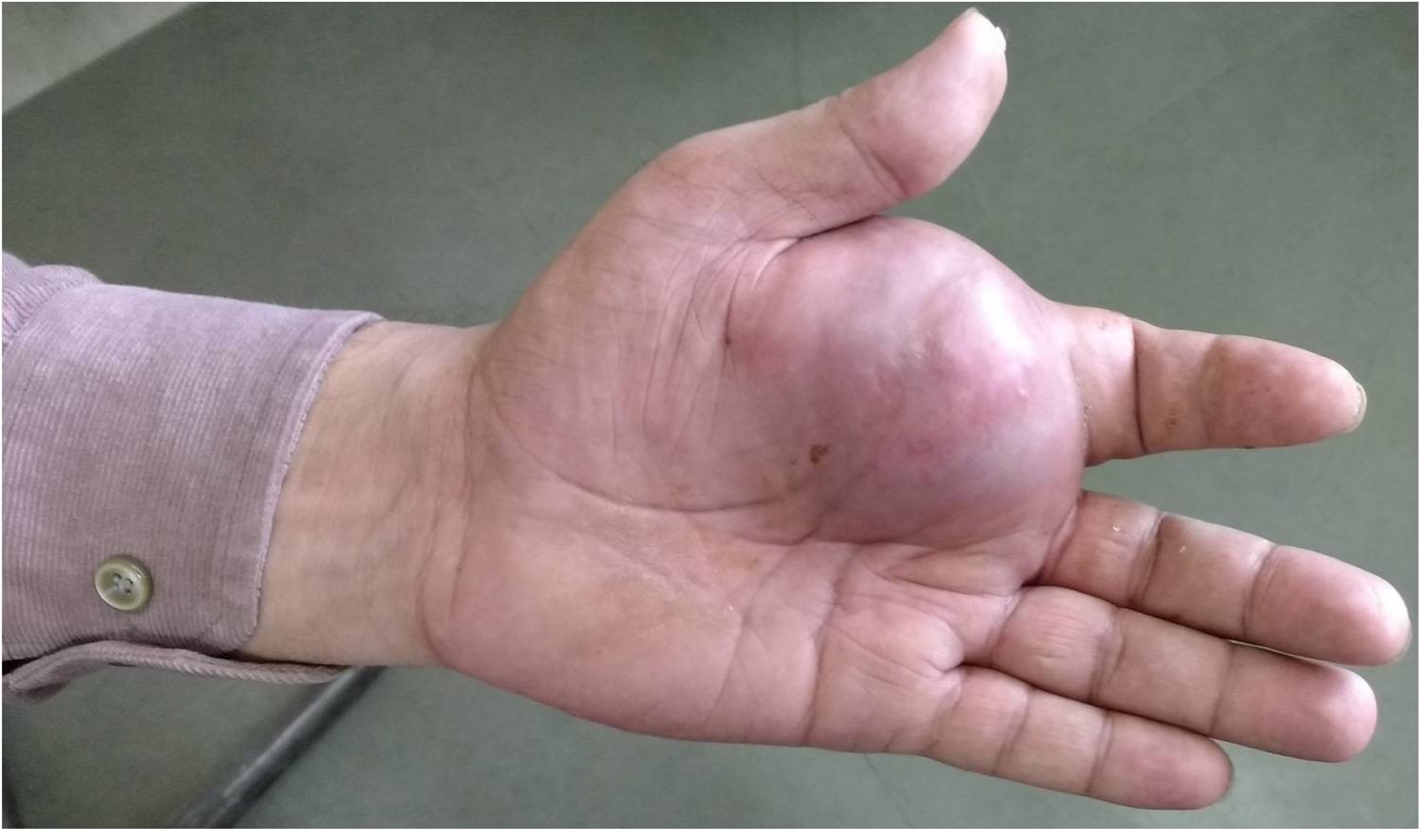
Clinical photograph of swelling in the left hand with redness of overlying skin

A 49-year-old Indian man presented with progressively increasing swelling in his left hand of 2 months’ duration, along with pain and redness of overlying skin (Fig. 1).

**Fig. 2:**
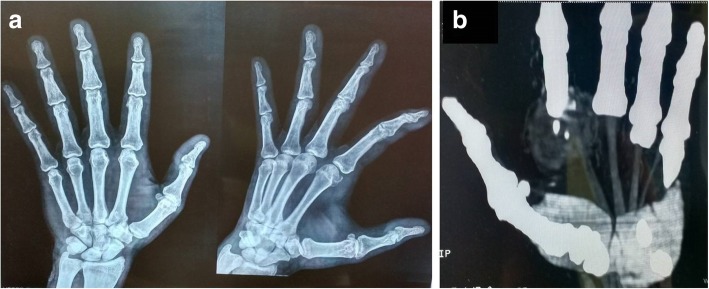
**a** Anteroposterior and lateral X-ray films of left hand showing a soft tissue lesion. **b** Contrast-enhanced computed tomography of left hand showing a soft tissue mass lesion, with peripheral enhancement and central necrotic areas in radial palmar soft tissue overlying second metacarpophalangeal region with no obvious bony osteolysis

There was no history of antecedent trauma. A detailed history revealed that he was diagnosed as having osteosarcoma of his right leg 3 years previously, for which he had an operation (wide local excision) and had received chemotherapy (MTX, doxorubicin), leading the tumor to resolve. Following this, sequential whole body PET scans were performed, which revealed no evidence of residual disease in his right leg or any tumor elsewhere. The remaining medical history, family history, and psychosocial history were unremarkable. On examination, the overlying skin was stretched and showed redness, and the swelling was hard in consistency. An X-ray showed a soft tissue lesion (Fig. 2a) Contrast-enhanced computed tomography (CECT) showed a soft tissue mass lesion of size 3.8 × 3 cm, with peripheral enhancement and central necrotic areas in radial palmar soft tissue overlying second metacarpophalangeal region with no obvious bony osteolysis (Fig. 2b).

**Fig. 3:**
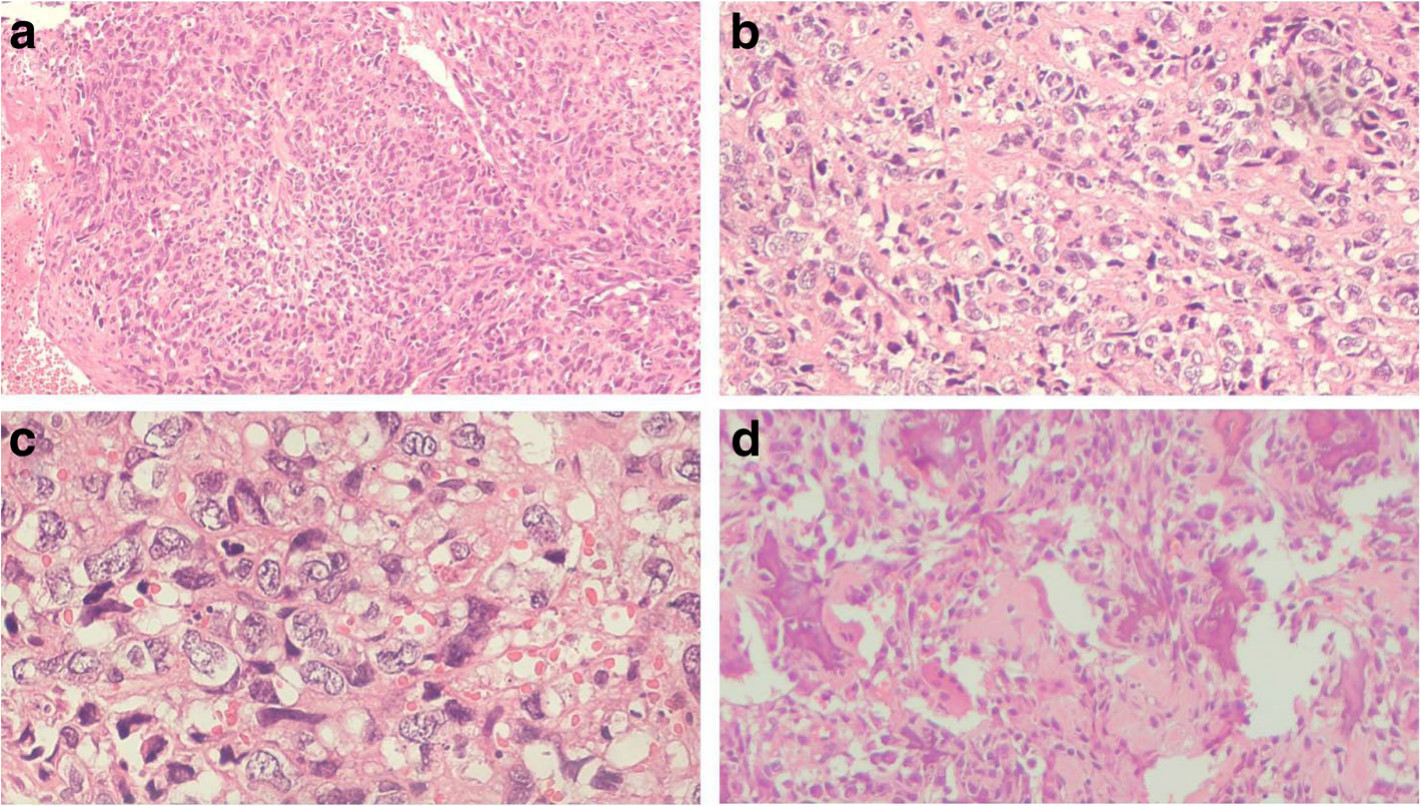
Hematoxylin and eosin sections showing a tumor with tumor cells arranged in sheets. Tumor cells are large, showing high degree of pleomorphism and atypia. They vary in shape from epithelioid to spindle to polygonal; they have eosinophilic cytoplasm. Nuclei have irregular nuclear membranes and prominent nucleoli: **a** × 4, **b** × 10, **c** × 20. **d** Hematoxylin and eosin section showing osteoid deposition, × 4

Possibilities of acute abscess, resolving hematoma, or aggressive soft tissue mass lesion were suggested. The aspirate from the swelling was sent for culture sensitivity which was sterile. An incision biopsy was performed. Hematoxylin and eosin (H&E) sections showed a tumor with tumor cells arranged in sheets and vague nodules, separated by large areas of hemorrhage and necrosis. The tumor cells were large, showing a high degree of pleomorphism and atypia, varying in shape from epithelioid to spindle to polygonal, and had eosinophilic cytoplasm with cytoplasmic vacuolation. Nuclei had irregular nuclear membranes and prominent nucleoli. A large number of atypical mitoses were seen. Numerous variable-sized blood vessels were seen showing presence of tumor emboli (Fig. 3a–c).

**Fig. 4:**
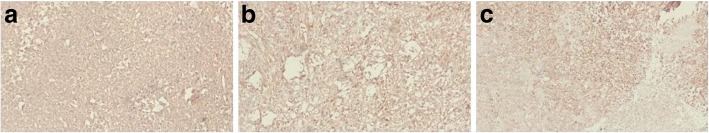
**a**–**c** Positive immunohistochemistry for cytokeratin, epithelial membrane antigen, and vimentin, respectively

**Fig. 5:**
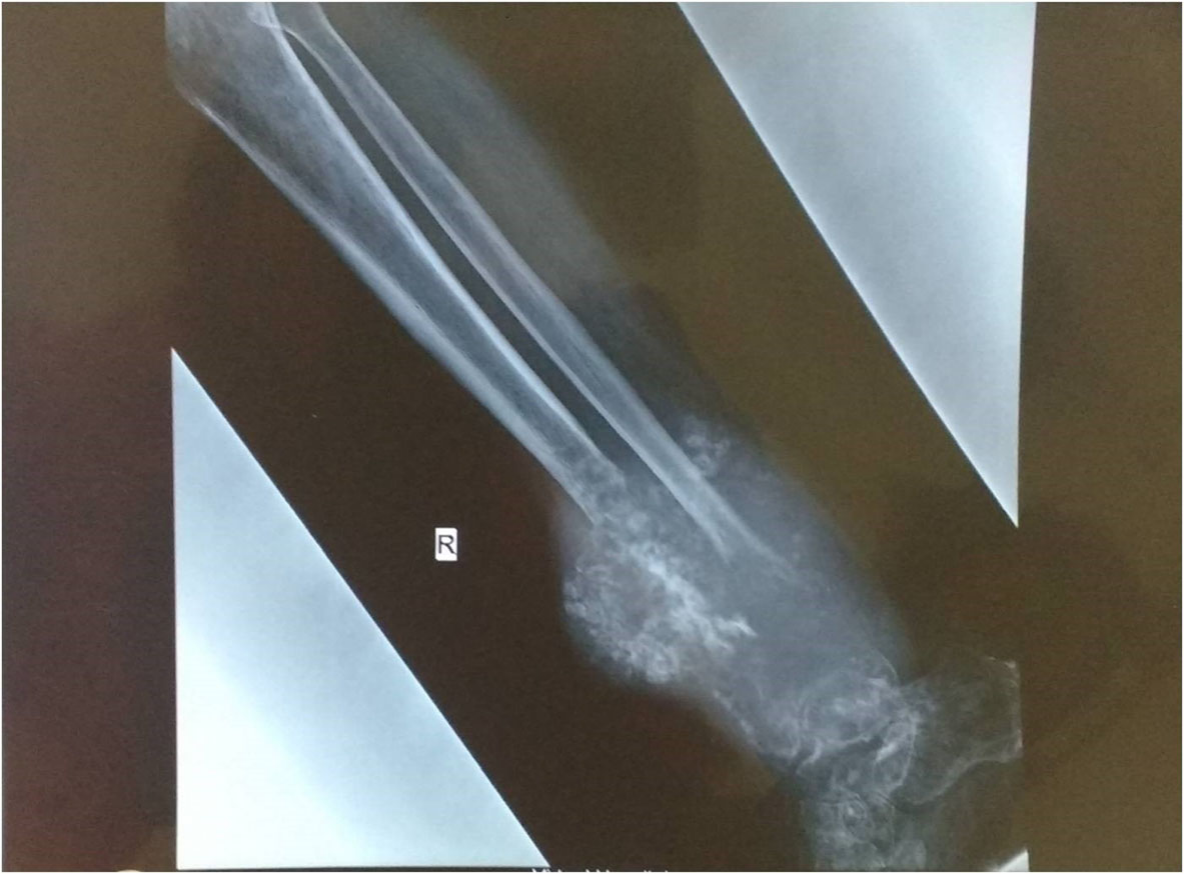
X-ray film of right leg showing large lytic expansile lesion involving middle and lower third of tibia and fibula

An immunohistochemistry (IHC) panel was put up, comprising cytokeratin (CK), epithelial membrane antigen (EMA), vimentin, osteopontin, smooth muscle actin (SMA), desmin, leukocyte common antigen (LCA), and cluster of differentiation (CD) 99 and CD 34. The tumor cells were positive for CK, EMA, osteopontin, and vimentin (Fig. 4a–c). They were negative for SMA, desmin, LCA, S-100, and CD 99 and CD 34, ruling out leiomyosarcoma, rhabdomyosarcoma, lymphoma, Ewing’s sarcoma, and angiosarcoma, respectively. Furthermore, on extensive sampling, sections from the tumor showed presence of osteoid (Fig. 3d). Previous radiology (Fig. 5) and histopathology were also reviewed (findings were similar to that of the present lesion) and the diagnosis of osteosarcoma was confirmed.

Based on history, radiology, morphology, and IHC, a final diagnosis of extraskeletal (soft tissue) epithelioid osteogenic sarcoma of the left hand occurring as a metachronous tumor 3 years after diagnosis of primary osteosarcoma in the right leg (Amstutz type IIIb) was given. A whole body PET scan revealed no evidence of viable residual/recurrent disease at our patient’s right leg; however, heterogeneously enhancing soft tissue mass lesion in thenar region of his left palm causing bony erosion of underlying second metacarpal bone, heterogeneously enhancing irregular mass lesion in upper lobe of his right lung, along with bilateral pulmonary nodular lesions were suggestive of metastatic deposits. Prior to the discovery of the metachronous tumor, his lungs and skeleton had been continually free of disease, and the lung lesion was attributed to the metachronous tumor. He underwent wide local excision of the palm tumor. This was followed by chemotherapy (MTX, doxorubicin, cisplatin) for the hand as well as the lung deposits. He has been on follow-up for the past 8 months, is still under follow-up, and is presently doing fine (Additional file 1).

## Discussion

Osteosarcoma is a malignant bone tumor, which affects the metaphysis of long bones predominantly [10]. Metachronous osteosarcoma is uncommon with a reported incidence of 1–10% of all cases of osteosarcoma [4]. The age group affected by metachronous osteosarcoma is usually adolescents and young adults; it presents as asymmetric metaphyseal lesions in long tubular bones. There is a slight male predominance among young patients (< 18-years old), and a 3:1 male predominance exists in adults (> 18-years old). The primary lesion occurs commonly at the distal end of the femur, proximal end of the tibia, proximal end of the femur, along with pelvis and proximal end of humerus [11].

The literature states two main debatable theories regarding the multifocality of osteogenic sarcoma. Whether these represent: (a) simultaneously arising multisite, synchronous, primary lesions; or (b) single-site origin, with one primary site followed by early, rapidly progressive metastatic lesions, is still a debatable issue. The argument in favor of multiple primary tumors is that osteosarcoma manifests as a single primary bone lesion that very rarely has bony metastasis [8]. On the other hand, there is also literature favoring multifocal tumors to be metastatic. In their study, Parham *et al.* [12] found no significant clinicopathological difference between pediatric patients with multifocal osteosarcoma presenting with or without pulmonary metastases. They concluded that absence of pulmonary metastasis is insufficient to prove the hypothesis of multiple primary origins. A similar opinion was held by Mohoney *et al*. [13], that metachronous multifocal osteosarcoma probably represents metastatic osteosarcoma to bone because pulmonary metastasis is frequently combined. However, some cases of metachronous multifocal osteosarcoma might represent new primary lesions occurring in damaged or dystrophic mesenchymal tissue which have a propensity to undergo sarcomatous degeneration at a later stage. Thus, in the present case, the two tumors may be independent primaries or metastatic lesions.

The most frequently used classification of multifocal osteogenic sarcoma is given by Amstutz [11], according to which the present case is type IIIb.

Epithelioid osteosarcoma is a rare type of osteosarcoma with only a few cases reported in the literature [14–19]. This lesion was first reported by Scranton *et al.* in 1975 [19], as having an “endocrine” pattern that shows pleomorphic epithelioid cells with malignant osteoid deposition. The age of the patients ranges from first to seventh decades, with males being affected more often than females (2:1), and the lesion occurring predominantly in the long bones [18, 20]. The present case is of a 49-year-old man.

Epithelioid osteosarcoma constitutes a diagnostic challenge because its histology mimics that of other malignant tumors that have epithelioid features. Metastatic carcinomas from breast, lungs, colon, or prostate stimulating osteoid formation may be misinterpreted as epithelioid osteosarcoma [21]. The patients affected by epithelioid osteosarcoma are usually ≥ 40 years of age [16]. Other tumors with a histological picture mimicking that of epithelioid osteosarcoma are metastatic melanoma, lymphoma, Ewing’s sarcoma, angiosarcoma, fibrosarcoma, leiomyosarcoma, rhabdomyosarcoma, and malignant peripheral nerve sheath tumor. In the present case, which is of a 49-year-old man who presented with a metachronous osteosarcoma occurring in the soft tissue of his left hand 3 years after the treatment of primary osteosarcoma in his right leg, those tumor types were excluded based on an integrated study of the clinical, radiologic, histopathologic, and immunohistochemical results.

Epithelioid osteosarcoma has an aggressive behavior and its prognosis is poor [18]. In a case series by Okada *et al.*, six cases of epithelioid osteosarcoma were reported, five of which died within 5 to 52 months of initial surgery or hospital admission [20].

This is probably the first reported case of extraskeletal (soft tissue) epithelioid osteosarcoma occurring as a metachronous tumor 3 years after diagnosis of primary osteosarcoma in the right leg. Our patient also had lung metastasis.

The interval between discovery of the primary tumor and the metachronous (first) tumors varied from 12 to 78 months (average, 39 months) [22],11 months to 7.33 years [1], and 9 months to 14 years [23] in various studies. In this case, it was 3 years.

It has been postulated that some pre-existing diseases, for example, Rothmund–Thomson syndrome, Paget disease, and Fanconi anemia, may have a role in the development of metachronous osteosarcoma [8, 24].

The use of multiple imaging modalities such as computed tomography (CT), MRI, and bone scan can lead to an improvement in detecting as well as evaluating the progression of the disease. Karyotyping may be used to reveal a clonal relation between these tumors to help in estimation of prognosis and in guiding treatment [9].

The prognosis of metachronous skeletal osteosarcoma is poor as compared to that of relapse limited to lungs. In late metachronous osteosarcoma, combined-modality therapy comprising surgery and aggressive chemotherapy may affect long-term survival. Thus, principles used for treatment of primary osteosarcoma may be applicable to late metachronous osteosarcoma [25].

In a large study, the survival of patients with metachronous osteosarcoma varied from 5 months to 11 years [23]. In another series, a correlation was found between survival and time taken for developing metachronous tumor [25]. The 5-year post-metachronous survival rate after combined modality therapy in patients who developed metachronous tumors ≥ 24 months and ≤ 24 months from diagnosis of the primary osteosarcoma was 61% and 8%, respectively. In the series of Jaffe *et al.*, 5 of 11 patients attained survival rates ranging from 18+ to 50+ months post detection of first metachronous tumor [3]. Their study indicated that lifelong follow-up of surviving patients with osteosarcoma is necessary and if metachronous osteosarcoma is discovered, it should be treated with curative intent [3].

## Conclusion

This is probably the first reported case of extraskeletal (soft tissue) epithelioid osteosarcoma of the left hand occurring as a metachronous tumor 3 years after diagnosis of primary osteosarcoma in the right leg. Therefore, if a patient with a previous history of osteosarcoma develops a new swelling, the possibility of a metachronous osteosarcoma should always be considered, even at unusual sites like soft tissue. This diagnosis has substantial significance since the prognosis of metachronous skeletal osteosarcoma is poor as compared to that of relapse limited to lungs. In late metachronous osteosarcoma, combined-modality therapy comprising surgery and aggressive chemotherapy may affect long-term survival. Thus, in view of this case and similar cases, lifelong follow-up of surviving patients with osteosarcoma is necessary and if metachronous osteosarcoma is discovered, it should be treated with curative intent.

## Additional file

Additional file 1: Case Timeline. (DOCX 14 kb)
