# Supplementary material for: Metachronous extraskeletal (soft tissue) epithelioid osteogenic sarcoma: a case report
Source: J Med Case Rep. 2019 May 9;13:136. doi: 10.1186/s13256-019-2070-3 (PMC6507060; doi:10.1186/s13256-019-2070-3)
Supplement: Supplementary file 1 — Case Timeline. (DOCX 14 kb) [file 13256_2019_2070_MOESM1_ESM.docx]

Osteosarcoma of right leg 3 years previously, resolved on operation & chemotherapy. The remaining medical history, family history and psychosocial history were unremarkable

Current Illness: 49 years old male presented with progressively increasing swelling in the left hand since 2 months, along with pain and redness.

Physical examination: overlying skin was stretched, showed redness, and swelling was hard.

Diagnostic Evaluations: CT scan revealed soft tissue mass lesion.

On biopsy differential diagnosis were leiomyosarcoma, rhabdomyosarcoma, lymphoma, Ewing’s sarcoma, angiosarcoma, osteosarcoma.

Osteoid confirmed osteosarcoma.

Final follow up: Presently doing well.

Whole body PET scan: lung metastasis

Initial Treatment: Wide local excision

Referral: medical oncologists

Adjuvant Chemotherapy

Final Diagnosis: Metachronous extraskeletal (soft tissue) epithelioid osteogenic sarcoma.

Resolution of this episode of care
